# Differential effects of the changes of LDL cholesterol and systolic blood pressure on the risk of carotid artery atherosclerosis

**DOI:** 10.1186/1471-2261-12-66

**Published:** 2012-08-17

**Authors:** Kuo-Liong Chien, Yu-Kang Tu, Hsiu-Ching Hsu, Ta-Chen Su, Hung-Ju Lin, Ming-Fong Chen, Yuan-Teh Lee

**Affiliations:** 1 Institute of Epidemiology and Preventive Medicine, College of Public Health, National Taiwan University, Taipei, Taiwan; 2 Department of Internal Medicine, National Taiwan University Hospital, Taipei, Taiwan; 3 Division of Biostatistics, Leeds Institute of Genetics, Health & Therapeutics, University of Leeds, Leeds, UK; 4 China Medical University Hospital, Taichung, Taiwan

**Keywords:** Latent growth curve modeling, Carotid intima media thickness, Blood pressure, LDL cholesterol

## Abstract

**Background:**

The effects of baseline and changes in blood pressure and low density lipoprotein (LDL) cholesterol on the carotid intima media thickness (IMT) have not been well documented.

**Methods:**

A total of 2572 adults (mean age 53.8 years, 54.6% women) in a Taiwanese community undertook three blood pressure and LDL cholesterol examinations over 6 years. Latent growth curve modeling was used to investigate the effects of baseline and change in blood pressure and LDL cholesterol on IMT.

**Results:**

Greater baseline LDL and blood pressure were associated with an increase in IMT (0.005 ± 0.002 mm per 1 mg/dL [p = 0.006] and 0.041 ± 0.004 mm mmHg [p <0.0001], respectively. Change in blood pressure was associated with a significant increase in IMT (0.047±0.016, P = 0.004), whilst the association between change in LDL and change in IMT was not statistically significant (0.008±0.006, P = 0.20).

**Conclusions:**

Carotid IMT was associated with baseline blood pressure and LDL cholesterol, yet only changes of blood pressure, not LDL cholesterol, were related to carotid IMT during the 6-year observation.

## Background

It has been suggested that carotid intima media thickness (IMT) is associated with metabolic syndrome components. One cross-sectional study on 2268 Americans showed that the average increase in IMT was 0.52 mm in people with no metabolic syndrome and 0.69 mm in people with 4 metabolic syndrome components [1]. However, the cross-sectional study design and the analysis of non-specific components provides limited evidence for a causal interpretation of these associations [1]. A randomized controlled trial which recruited more than 19000 adults in Europe showed that a reduction in both lipids and blood pressure decreased the risk of cardiovascular events [2], indicating that blood pressure and lipids may play an important role in the risk of atherosclerosis.

IMT of the common carotid artery are considered useful indicators of carotid atherosclerosis [3-5]. Al-Shali and colleagues [6] found that age and hypertension were associated with carotid IMT, whereas total cholesterol, current smoking, diabetes, and gender were not. It is noted that most studies have not distinguished differences in blood pressure and lipid profiles [5,7]. Evidence on the cumulative effects of blood pressure and cholesterol is insufficient. Some studies have shown that carotid plaque score is associated with an increased risk of cardiovascular disease [7-11], but results from clinical trials on the relationship between the reduction in blood pressure and cholesterol and change in IMT are inconsistent [12-14]. Previous controlled trials showed that except diuretics, blood pressure lowering medication and statin decreased IMT modestly [15,16], whilst the associations of blood pressure and LDL cholesterol with carotid IMT remain unclear. It would therefore be useful to look into the effects of changes in repeated blood pressure and cholesterol measurements on IMT in longitudinal cohorts. The aim of this study is to use repeated measurements of blood pressure and LDL cholesterol levels over 6 years to investigate the effects of changes in blood pressure and cholesterol on carotid IMT among Taiwanese adults in a community cohort.

## Methods

### Study design and participants

Details of this cohort have been reported elsewhere [17-19]. Briefly, the Chin-Shan Community Cardiovascular Cohort Study (CCCC) started in 1990 by recruiting 1703 men and 1899 women of Chinese ethnicity aged 35 years old and above from the Chin-Shan township, 30 km north of metropolitan Taipei, Taiwan. Information about the lifestyle and medical conditions were collected with questionnaires from in-person interviews every 2 years for the initial 6 years up to 1994-1995. The validity and reproducibility of these measurements have been published previously [19]. A total of 2244 participants had complete carotid ultrasonography measurements in 1994-1995 after incomplete data of blood pressure, lipid (n=328), and incomplete carotid ultrasonography (n=1030) were excluded (Additional file 1: Table S1). The National Taiwan University Hospital Institutional Review Board approved the study protocol, and the informed consent for participation in the study was obtained from each participant.

### Clinical measurements and biochemical markers

Body mass index was calculated as weight (in kilograms) over height (in meters) squared. Blood pressure was measured twice on the right arm supported and positioned at the level of the heart with a mercury sphygmomanometer, after participants were seated comfortably for ten minutes. All participants were asked to refrain from smoking cigarettes for at least 30 minutes and from drinks with caffeine before examination.

Hypertension was defined according to the criteria established by the Seventh Joint National Committee [20]: systolic blood pressure more than or equal to 140 mmHg or diastolic blood pressure more than or equal to 90 mmHg; or a history of taking hypertension medications.

Repeated measurements of blood pressure and lipid profiles were collected biannually from 1990, and the procedures for taking blood samples have been reported elsewhere [21,22]. Briefly, all venous blood samples drawn after a 12-hour overnight fast were immediately refrigerated and transported within 6 hours to the National Taiwan University Hospital. Serum samples were then stored at -70°C before biochemical analysis of total cholesterol, triglycerides, and high density lipoprotein (HDL) cholesterol. Standard enzymatic tests for serum cholesterol and triglycerides were used (Merck 14354 and 14366, Germany, respectively). HDL levels were measured in supernatants after the precipitation of specimens with a magnesium chloride phosphotungstate reagent (Merck 14993). LDL cholesterol concentrations were calculated as total cholesterol minus cholesterol in the supernatant by a precipitation method (Merck 14992) [23].

### Ultrasound imaging on carotid artery measurements

The procedures for ultrasound sonography of carotid arteries have been reported elsewhere [17,24], and the recommendation from the expert recommendation was strictly followed [25]. In brief, IMT were measured by using a Hewlett-Packard SONO 1500 ultrasound system, equipped with a 7.5 MHz real-time B-mode scanner. Patients were asked to lie supinely with the neck extended in a slightly lateral rotation. We then scanned the carotid artery and identified the lumen of the carotid artery beneath the surface of the neck. IMT was defined as the distance between the front edge of the first echogenic line (lumen-intima interface) and the front edge of the second line (media-adventitia interface) in the far wall of the vessel. The same procedure was undertaken on the other side of the neck. The measurement of IMT was made on the ECG-gated R-wave (diastole) phase using a semiautomated technique. Two measures were made on each side. The measurement of IMT over the distal 1cm of the common carotid artery was noted [24]. The maximum IMT was defined by averaging maximum measurements on both sides [26]. Participants’ medical status was unknown to cardiologists who carried out the examinations. The inter-examiner correlation coefficients ranged from 0.86 to 0.93, and the intra-examiner correlation coefficients ranged from 0.70 to 0.87 for both sides of common carotid artery IMT measurements. We excluded participants with IMT more than 1.5 mm and those with significant plaque formation.

### Statistical analysis

Continuous variables are presented as mean and standard deviation, and categorical variables in contingency tables. We used a linear regression model to test the associations of repeated systolic blood pressure and LDL with the carotid IMT. We used the diagnostic tools for collinearity, namely the tolerance and variance inflation factors, to evaluate the multicollinearity problem in our model [27].

**Figure 1:**
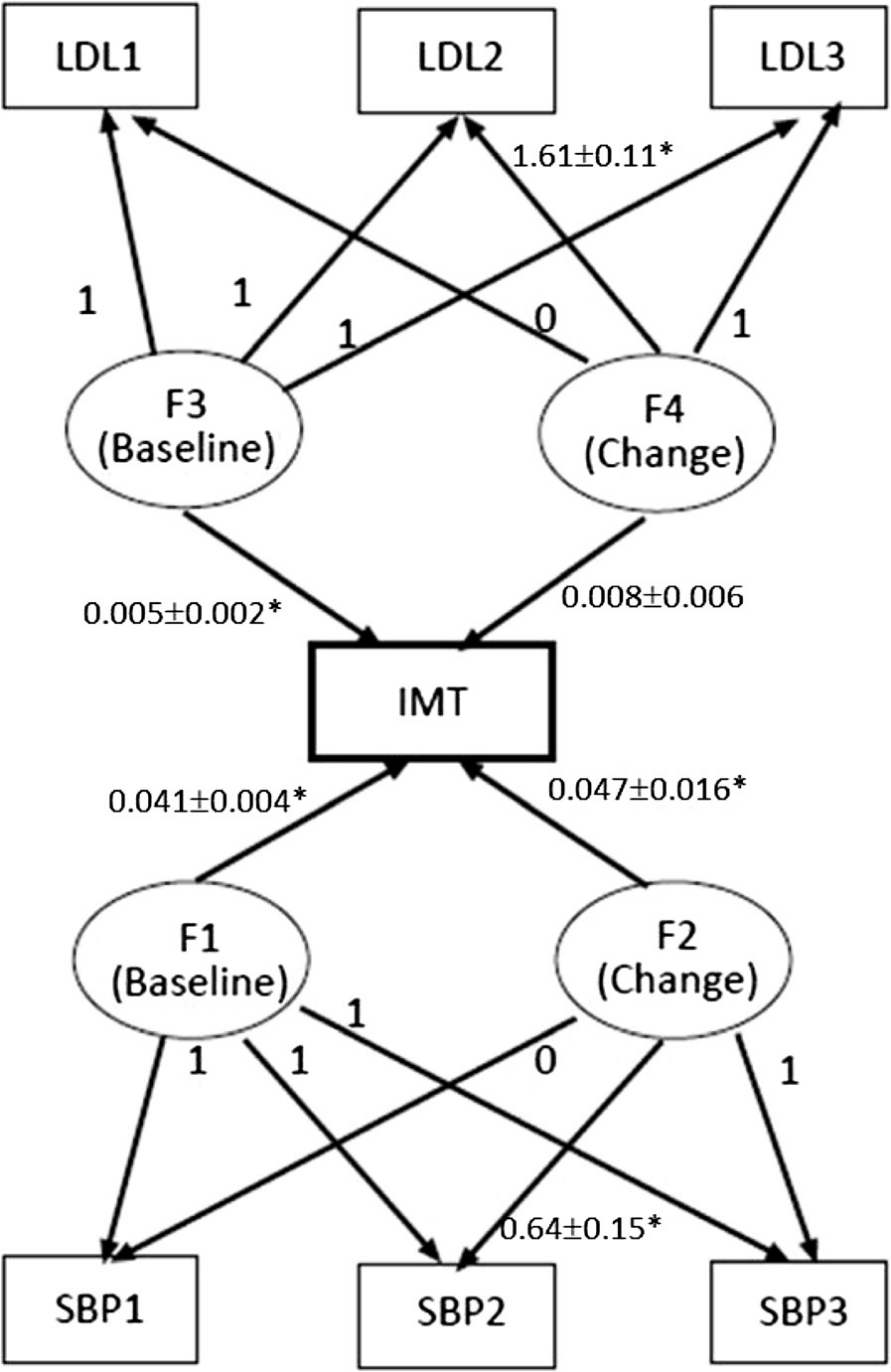
**Path diagram for bivariate latent growth curve modeling to estimate the effects of SBP and LDL on IMT within the same model.** The three repeated measurements of systolic blood pressure (SBP1, SBP2, and SBP3), the three repeated measurements of LDL cholesterol (LDL1, LDL2, and LDL3), and carotid IMT (IMT) are in squares. F1 and F2 are the estimated baseline SBP and overall change in SBP, respectively; and F3 and F4 the estimated baseline LDL and changes in LDL, respectively. To simply the presentation, we did not show the residual error terms and the correlations among the later variables in the graph. The estimated coefficients and standard errors were shown and the significance level <0.05 was presented as ‘*’. Footnote: the estimated means±standard errors for F1 (SBP intercept) were 47.7±3.2, F2 (SBP change) were 17.2±3.3, F3 (LDL intercept) were 22.1±7.6, F4 (LDL change) were 23.6±5.4, all significance levels <0.05.

Latent growth curve modeling, a special application of structural equation modeling (SEM), was then used to analyze the associations between changes in LDL cholesterol and systolic blood pressure (SBP) over the 6-year period and final IMT. Separate non-linear latent growth curve model analyses for LDL cholesterol and carotid IMT and for systolic blood pressure were undertaken [28]. Figure 1 shows the path diagram for the bivariate latent growth curve model. Briefly, one part of latent growth curve models used two latent variables to estimate baseline SBP and LDL and their changes over 6 years (F1 & F2 for SBP and F3 & F4 for LDL in Figure 1). For example, in the SBP model, the three observed SBP can be expressed as three linear regression models of F1 and F2, and the coefficients are regression weights, also known as factor loadings, for F1 and F2. When the first coefficient fixed to equal 0 and third coefficient fixed to unity, F1 becomes the estimated baseline SBP, and F2 becomes the overall change in SBP. If velocity of change in SBP is constant, we can fit a linear growth curve model by fixing the second coefficient to equal 0.5 (because the three SBP were measured every 2 years). In other words, latent growth curve modeling aims to estimate baseline values and changes from baseline using the observed repeated measurements. One advantage of this approach is to reduce the number of variables in the model and to overcome the problem with collinearity. From a statistical point of view, latent growth curve modeling is equivalent to multilevel modeling for longitudinal data, and a non-technical introduction to latent growth curve modeling can be found in our previous publication [29]. We can also allow the factor loading for intermediate measurements to be freely estimated; if the changes in LDL or systolic blood pressure were linear, the factor loading for the intermediate measurement would be close to 0.5. The advantages of this approach are that a nonlinear change pattern can be estimated without using polynomial functions, and the means or intercept for the latent variables for changes (i.e. F2) can be directly interpreted as the total changes from baseline over the observation period. Age at baseline, sex, and body mass index were adjusted as confounders.

Next, a bivariate latent growth curve model was then constructed in which the IMT was regressed on the baseline and changes of both LDL cholesterol and systolic blood pressure. Covariates, including age, gender, and baseline body mass index were also included in the final model. IMT values were multiplied by 100 to prevent rounding errors in the computation. Several statistics were evaluated for the overall model fit: (1) a small chi-square value for model fit relative to the model degrees of freedom with a *P* value greater than 0.05 indicated that the proposed model fitted the data relatively well; (2) a comparative fit index (CFI) greater than 0.95 indicated a good model; (3) a Root Mean Square Error of Approximation (RMSEA) value less than 0.05 was considered as a good model; and (4) a Standardized Root Mean Square Residual (SRMR) less than 0.1 indicated a good model fit [30]. All statistical analyses were performed with the statistical software packages SAS version 9.1.3 and Mplus version 5 [31].

## Results

A total of 2244 adults (mean age of 54.3 years; 1238 women) were recruited into this study. Table 1 provides a summary of three repeated measurements of blood pressure, LDL cholesterol and carotid IMT. LDL cholesterol decreased in later examinations, yet non-HDL cholesterol and systolic blood pressure values increased progressively. The prevalence rates of elevated LDL level (>=220 mg/dL) in the study participants were 2–4% and the rates of high blood pressure (>=140 mmHg) were 20–24%.

**Table 1:** Basic characteristics of three repeated measurements of blood pressure and lipid profiles, and carotid intima media thickness values in the study participants who provided completed carotid data(n = 2244)

	**N**	**%**
Gender		
Women	1238	55.2
Men	1006	44.8
Current smoking	751	33.5
	**Mean**	**SD**
Age, years	54.3	11.7
Body mass index, kg/m^2^	23.7	3.4
1st LDL cholesterol, mg/dL	139.8	43.8
2nd LDL cholesterol, mg/dL	123.0	43.5
3rd LDL cholesterol, mg/dL	129.9	39.4
1st Systolic BP, mmHg	125.0	20.2
2nd Systolic BP, mmHg	125.6	19.9
3rd Systolic BP, mmHg	126.0	19.4
1st HDL cholesterol, mg/dL	47.0	12.2
1st fasting glucose, mg/dL	109.8	30.6
Carotid intima media thickness, mm	0.74	0.24

Abbreviations: *LDL*, low density lipoprotein; *HDL*, high density lipoprotein; BP, blood pressure.

Using a multiple linear regression model (Table 2), we found that age, gender and the first and second systolic pressure was modestly associated with carotid IMT (*P <*0.05). However, due to the problem with high correlations amongst the explanatory variables, the three LDL cholesterol and the third systolic blood values had non-significant associations with the carotid IMT. The regression diagnostic tests, including variance inflation factors and collinearity diagnostic statistics, showed substantial collinearity among the variables.

**Table 2:** The estimated coefficients, standard errors, and significance levels of repeated measurements of systolic blood pressure and LDL-C levels for the carotid IMT magnitude using traditional multiple linear regression model in the study participants

**Covariates**	**Unit**	**Estimate**	**Standard error**	**P**
Intercept		−1.76	0.58	0.003
Age	+1 yr	0.086	0.004	<.0001
Gender	Men vs. women	0.56	0.13	<.0001
Body mass index	+1 kg/m^2^	−0.018	0.015	0.23
Smoking status	Yes vs. no	0.316	0.135	0.019
1st HDL cholesterol	+1 mg/dL	−0.004	0.004	0.28
1st fasting glucose	+1 mg/dL	0.002	0.002	0.18
1st Systolic BP	+1 mmHg	0.009	0.003	0.007
2nd Systolic BP	+1 mmHg	0.017	0.003	<.0001
3rd Systolic BP	+1 mmHg	0.005	0.003	0.16
1st LDL cholesterol	+1 mg/dL	0.001	0.001	0.39
2nd LDL cholesterol	+1 mg/dL	0.001	0.001	0.37
3rd LDL cholesterol	+1 mg/dL	0.003	0.002	0.07

*Abbreviations*: *BP*: blood pressure, *LDL*: low density lipoprotein.

Results from the bivariate latent growth curve model are shown in Figure 1. The estimated factor loading for the intermediate measurement was 1.61 for LDL cholesterol and 0.62 for systolic blood pressure change, indicating non-linear increases in repeated LDL cholesterol and systolic blood pressure during the observation period. In addition, one unit increase of baseline LDL (mg/dL) and blood pressure (mmHg) was associated with a 0.005 mm (95% confidence interval [CI]: 0.001 to 0.009) and 0.041 mm (95% CI: 0.033 to 0.049) increase in carotid IMT, respectively. An increase in blood pressure was significantly associated with a modest increase in IMT (0.047 per 1 mmHg; 95% CI: 0.015 to 0.079, *P =* 0.004). However, the positive association between the increase in LDL and carotid IMT was not statistically significant (0.008 per 1 mmHg; 95% CI: -0.004 to 0.020, *P =* 0.20). The final model fit the data well; the chi-square (DF = 15) value was 21.2 with the p-value as 0.13, indicating the relations amongst the variables in the model were consistent with (non-sig different from) the observed data.

## Discussion

In this Taiwanese cohort, we found associations of carotid IMT with blood pressure and LDL cholesterol. When their effects were taken into account simultaneously, the positive association between the increase in LDL cholesterol and IMT became attenuated and non-significant. This indicates that change in systolic blood pressure may be a better predictor or a stronger risk factor for IMT.

The effects of blood pressure and lipids on carotid IMT have been reported in previous studies [1,32]. In one study based on 2268 adults who undertook health examinations, increased numbers of metabolic risk factors were associated with an increased IMT in different ethnic groups [1]. Another study based on 1809 young Finnish adults also showed that metabolic components were significantly associated with carotid IMT, and the progress of IMT among these young adults was related to obesity, LDL and insulin resistance [32]. Another small study in Italy (240 healthy adults) from health checkups also showed an additive synergistic effect of metabolic components for IMT [33]. Exploratory factor analysis was used to extract three factors; obesity/dyslipidemia, hypertension, and hyperglycemia [33]. However, the specific effects of the components were unclear in the factor analysis strategy. In addition, these studies were cross-sectional in design, without considering the cumulative effects of both blood pressure and lipids on carotid atherosclerosis. Our findings from the multiple linear regression model showed similar findings to those in the study of Al-Shali and colleagues [6]. In this study, we used latent growth curve modeling to investigate the associations of both baseline and changes in blood pressure and lipids on carotid IMT. Change in blood pressure seems to have a stronger effect on IMT than change in LDL cholesterol, suggesting the effects of lipid-lowering treatment on carotid IMT regression might be small [34]. This is consistent with the findings from clinical trials on lipid-lowering therapies in which medications for reducing LDL had inconsistent effects on IMT, as shown by numerous statin studies including METEOR (rosuvastatin) [35], ACAPS (lovastatin) [36], KAPS (pravastatin) [37], PLAC-II (pravastatin) [38], BCAPS (fluvastatin) [39], FAST (pravastatin) [40], and REGRESS (pravastatin) [41]. The stronger association between changes in blood pressure and IMT may be because the hemodynamic change of blood pressure had a stronger impact on carotid intima media thickness, while injuries caused by LDL changes affected the carotid IMT slowly and did not manifest during the study period. Consequently, the change in IMT caused by carotid blood flow may be an important indicator for endothelial function. Evidence from antihypertensive clinical trials showed that change of BP was related to the severity of carotid IMT regression, and results from our observational study seem to support the same hypothesis that carotid IMT is primarily a mechanism of medial hypertrophy.

Evidence from randomized controlled trial data also showed differential effects of blood pressure and cholesterol lowering on carotid atherosclerosis. Among 508 combined hypertensive and hypercholesterolemic patients under a randomized control trial for 2.6 years, the progression of the carotid IMT were not significant for patients treated with fosinopril (−0.002±0.004 mm) or those with fosinipril and prastatin (−0.002±0.004 mm) [15]. Adding LDL cholesterol lowering drugs did not seem to affect the change of IMT under the blood pressure lowering effects, which was also consistent with our findings. Antihypertensive drugs were related to the carotid IMT progression or regression, and the mechanism is complex. For example, the net arterial volume expansion developed as a decrease in carotid IMT after antihypertensive medication. Therefore, further investigation on different mechanisms of carotid IMT progress by blood pressure lowering drugs was warranted.

Latent growth curve modeling is a new tool to biomedical and epidemiological research, while it has been widely used in the social sciences. Using software packages for structural equation modeling, latent growth curve modeling provides a flexible statistical framework for the analysis of repeated measures in clinical practice to investigate the baseline and changes of exposure to outcomes [29,42]. The main advantage of using latent growth curve modeling instead of traditional approaches is that repeated measurements of multiple variables (e.g. LDL and systolic blood pressure in this study) can be incorporated into one single model, and their association with a distant outcome (e.g. IMT) can then be tested. This method has been successfully applied in psychology and sociology [43], and could be a very useful tool for longitudinal data analysis in medical research [44,45]. In this study, we used latent growth curve modeling to estimate the effects of baseline values and changes from baseline on the carotid IMT. The clinical implication of our findings was two fold: firstly, baseline blood pressure and lipids are risk factors for carotid atherosclerosis severity; and secondly, keeping optimal blood pressure levels may be helpful for reducing carotid IMT.

Several limitations of our study should be mentioned. First, IMT, rather than plaque status, was the outcome for our investigation. Previous studies have shown that the numbers of plaque were related to cardiovascular complications [46] and events [47]. Second, we did not include the medications for lipid and blood pressure in our analysis because that information was not available. Third, we did not measure mean carotid IMT in the study participants, because our protocol was designed to measure maximal carotid IMT values [24]. Finally, carotid IMT magnitude was measured once and we did not estimate the progress of the IMT.

## Conclusions

In conclusion, our study found that carotid IMT was associated with baseline blood pressure and LDL cholesterol measurements, but only changes in blood pressure, not LDL cholesterol, were related to carotid IMT in this Taiwanese cohort.

## Competing interests

All authors declare no competing interests for preparing the draft.

## Authors’ contributions

KLC carried out the design and data collection and drafted the manuscript. YKT carried out statistical analysis and revised the draft. HCH participated in laboratory data quality control and biochemical analysis. TCS participated in collecting data and maintaining the study. HJL participated in collecting data. MFC and YTL conceived of the study, and participated in its design and coordination and helped to draft the manuscript. All authors read and approved the final manuscript.

## Pre-publication history

The pre-publication history for this paper can be accessed here:

http://www.biomedcentral.com/1471-2261/12/66/prepub

## Supplementary Material

Additional file 1 Table S1: Scheme for the recruitment of the study participants in this study. Click here for file
