# Supplementary material for: Differential effects of the changes of LDL cholesterol and systolic blood pressure on the risk of carotid artery atherosclerosis
Source: BMC Cardiovasc Disord. 2012 Aug 17;12:66. doi: 10.1186/1471-2261-12-66 (PMC3445849; doi:10.1186/1471-2261-12-66)
Supplement: Additional file 1 — Table S1: Scheme for the recruitment of the study participants in this study. [file 1471-2261-12-66-S1.docx]

**Supplementary Materials**

**Supplementary Table 1: Scheme for the recruitment of the study participants in this study**

*
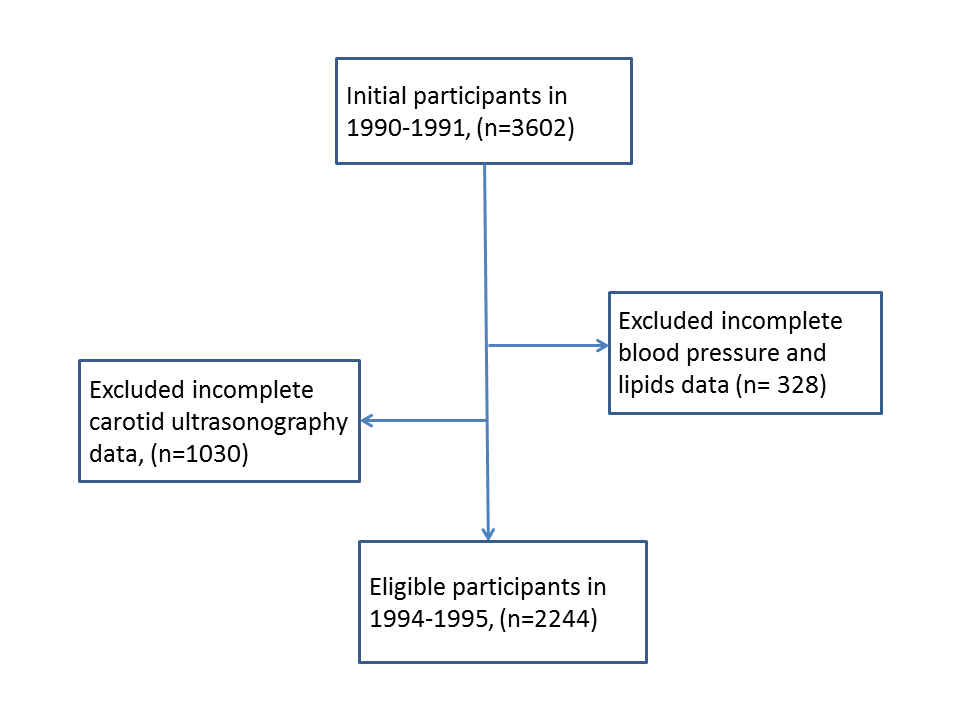
*
